# Hybridization and introgression during density‐dependent range expansion: European wildcats as a case study

**DOI:** 10.1111/evo.13704

**Published:** 2019-03-06

**Authors:** Claudio S. Quilodrán, Beatrice Nussberger, Juan I. Montoya‐Burgos, Mathias Currat

**Affiliations:** ^1^ Laboratory of Anthropology, Genetics and Peopling History, Department of Genetics and Evolution, Anthropology Unit University of Geneva Geneva Switzerland; ^2^ Laboratory of Vertebrate Evolution, Department of Genetics and Evolution University of Geneva Geneva Switzerland; ^3^ Department of Zoology University of Oxford Oxford United Kingdom; ^4^ Institute of Evolutionary Biology and Environmental Studies University of Zurich Zurich Switzerland; ^5^ Institute of Genetics and Genomics in Geneva (IGE3) Geneva Switzerland

**Keywords:** Density‐dependent dispersal, *Felis silvestris*, gene flow, invasive species, spatially explicit simulation

## Abstract

Interbreeding between historically allopatric species with incomplete reproductive barriers may result when species expand their range. The genetic consequences of such hybridization depend critically on the dynamics of the range expansion. Hybridization models during range expansion have been developed but assume dispersal to be independent from neighboring population densities. However, organisms may disperse because they are attracted by conspecifics or because they prefer depopulated areas. Here, through spatially explicit simulations, we assess the effect of various density‐dependent dispersal modes on the introgression between two species. We find huge introgression from the local species into the invasive one with all dispersal modes investigated, even when the hybridization rate is relatively low. This represents a general expectation for neutral genes even if the dispersal modes differ in colonization times and amount of introgression. Invasive individuals attracted by conspecifics need more time to colonize the whole area and are more introgressed by local genes, whereas the opposite is found for solitary individuals. We applied our approach to a recent expansion of European wildcats in the Jura Mountains and the hybridization with domestic cats. We show that the simulations explained better the observed level of introgression at nuclear, mtDNA, and Y chromosome markers, when using solitary dispersal for wildcats instead of random or gregarious dispersal, in accordance with ecological knowledge. Thus, use of density‐dependent dispersal models increases the predictive power of the approach.

Organisms may expand or shift their geographical range as a consequence of translocation, modifications of habitat, and climate change (e.g., Brown et al. 2010). Among the interactions that take place during range expansions, interspecific hybridization is of growing concern in both evolutionary and conservation biology. This is the consequence of the emergence of new overlaps in breeding period and location, leading to hybridization between historically allopatric species with incomplete reproductive barriers and breaking their independent evolution (Arnold and Martin 2010).

The resulting introgression between the interacting species is influenced by the dynamic of range expansion (e.g., Johannesen et al. 2006; Garcia et al. 2011; Ren et al. 2012). The invasive species usually arrives with few individuals, whereas the local species may be considered to be at demographic equilibrium. This demographic imbalance at the wave front of the invasive range expansion results in asymmetric introgression between species, with a larger gene flow from the local to the invasive than the reverse. This asymmetrical pattern was demonstrated by Currat et al. (2008) by using genetic simulations and an extensive literature survey, and since been demonstrated in a variety of organisms (e.g., Neiva et al. 2010; Mastrantonio et al. 2016; Garcia‐Elfring et al. 2017).

Models aiming at studying the genetic consequences of species range expansion have been developed, but assume dispersal to be independent from local population densities (Currat et al. 2008; Excoffier et al. 2014). This is not necessarily true for species that use information from conspecifics as attracting or repulsive signals for entering a given area (Quilodrán et al. 2014b). Density‐negative dispersal, or avoidance of populated areas, may be observed in solitary organisms with aggressive interactions (e.g., Aguillon and Duckworth 2015), whereas density‐positive dispersal, or attraction, may be observed in gregarious organisms with colonial behavior (e.g., Szostek et al. 2014). The classical model of density‐dependent distribution considers territorial species as avoiding conspecifics (Fretwell and Lucas 1969), but alternative examples showed that territorial organisms can also be attracted, for instance in migratory songbirds looking for suitable patches for survival or mating (Rushing et al. 2015). Previous studies have highlighted the critical importance of incorporating density‐dependent dispersal when investigating the consequence of species range expansion (Altwegg et al. 2013; Bocedi et al. 2014; Ponchon et al. 2015). Those studies achieve a better fit for the speed of colonization and a better prediction of population responses to environmental changes by incorporating density‐dependent dispersal. However, this body of work focused on the ecological implications and did not consider the genetic consequences of density‐dependent dispersal. Here, in a first part, we assess the influence of positive and negative density‐dependent dispersal during range expansion on the level of genetic introgression between interbreeding native and invasive organisms. We compare our results to those previously published using a dispersal model independent of local densities under the same simulation framework (Currat et al. 2008). Our main goal was to investigate how different modes of dispersal may affect the general pattern of neutral introgression reported after the expansion of an invasive species into the range of a local one.

In a second part, we exemplified the importance of incorporating density‐dependent dispersal in models of genetic introgression during range expansion by using the interactions between European wildcats (*Felis silvestris silvestris*) and domestic cats (*Felis s. catus*) as a case study. The European wildcats were widely distributed across Europe (Sommer and Benecke 2006), but habitat loss and hunting reduced their populations to near extinction during the 19th and 20th centuries (Stahl and Artois 1994). Conservation actions since then helped to increase the population in some parts of Europe (e.g., Say et al. 2012). The European wildcat is still listed as an endangered species in the Red List of various countries, the major threat being hybridization with domestic cats (Yamaguchi et al. 2015). Here, we restrict our analysis to the Jura region, in Switzerland, where wildcats were virtually extinct in the middle of 20th century, with no observations of individuals between 1943 and 1968 (Nussberger et al. 2007). The Swiss federal hunting law protected this species in 1962 (Duelli and Agosti 1994) and new observations in the Jura Mountains have been made since then. Several observations have been registered in the 1990s (Dötterer and Bernhart 1996), with an increasing trend in recent years (Nussberger et al. 2014), evidencing a range expansion of wildcats. A genetic characterization of the interbreeding between both cats also supports a recent increase of wildcat number in this area (Nussberger 2013; Nussberger et al. 2013; Nussberger et al. 2014). In a recent study, Nussberger et al. (2018) used spatially explicit simulations to show that a range expansion of wildcats during the last 50 years, involving hybridization with domestic cats, explains better the observed patterns of introgression compare with other possible scenarios noninvolving wildcat spatial expansion. This example illustrates the fact that in contrast to the usual situation with an expanding invasive species and a native local species, the situation can be reversed in cases where threatened species recolonize formerly occupied habitat after successful conservation actions are taken. Hence, in the case of the wildcat, the expanding species is native (wildcats), whereas the local species is nonindigenous (domestic cats). However, the dispersal of wildcats was assumed to be density independent, which is questionable due to their territorial behavior (Corbett 1979; Sunquist and Sunquist 2002; Biró et al. 2004). Here we extended this analysis by incorporating density‐dependent dispersal into the simulation framework designed by Nussberger et al. (2018) to investigate (1) which spatial dispersal behavior explains best this recent range expansion and the associated genetic evidence of introgression; and (2) if modeling density‐dependent dispersal improves the accuracy of predictions of the extent of introgression under range expansion.

## Methods

### SIMULATION OF RANGE EXPANSION WITH HYBRIDIZATION

To simulate the range expansion of an invasive species into an empty area or into a habitat already occupied by another local species, with or without genetic admixture, we used a modified version of the program SPLATCHE2 (Ray et al. 2010). This software was designed to simulate neutral genetic diversity in a spatially explicit landscape (Currat et al. 2004). Each simulation is divided in two consecutive steps: (1) a forward simulation of population demography and migration and (2) a backward coalescence simulation, conditioned on the demographic and migratory information of the first step, to reconstruct the genealogy of a series of samples. This genealogy was then used to compute the proportion of introgressed genes between species following the procedure described in Currat et al. (2008).

SPLATCHE2 simulates the evolution of a series of interconnected demes during a given number of generations. The distribution range of a single species is represented as one grid of demes arranged in a stepping‐stone manner, while two different species are represented by two superimposed grids, one for each of them. Gene flow between neighboring demes belonging to the same grid represents migration and is regulated by the parameter *m* (migration rate), whereas gene flow between superimposed demes belonging to different grids represents hybridization between species and is regulated by the parameter γ (interbreeding success rate). Within each deme, population density is logistically regulated using the parameters *r* (growth rate) and *K* (carrying capacity). Interspecific competition between two simulated species can be incorporated using the Lotka–Volterra equation (Volterra 1928; Lotka 1932). More information about algorithms is available in Ray et al. (2010), but the admixture model is the one improved by Excoffier et al. (2014) and is implemented as follows.

Admixture between species is simulated in every deme, in which *N_i_* and *N_j_* are diploid population sizes of the two species in the previous generation (*t* – 1). In the current generation (*t*), considering panmictic reproduction within demes, within and between species, newborn individuals ${\dot{N}}_{i}$ have a probability $N_{i}/\left(N_{i}+N_{j}\right)$ to have parental ascendance from the same species *i* and a probability $N_{j}/\left(N_{i}+N_{j}\right)$ to be a hybrid, with one parent coming from the species *j*. Therefore, the expected number of admixtures resulting in a transfer of genes *j* into the species *i* is $A_{ji}=\gamma {\dot{N}}_{i}N_{j}/\left(N_{i}+N_{j}\right)$. The transfer of genes *i* into the species *j* is driven by the same equation but inversing the equation subscripts. The value of γ thus measures the strength of barriers to gene flow between both species. A value equal to zero indicates no interbreeding between both species, whereas a value of 1 indicates that the reproduction is random between the species and they behave as a panmictic population. Any intermediate value implies that mating is nonrandom between both species (Quilodrán et al. 2014a, 2018a).

### NEW MODELS OF DENSITY‐DEPENDENT DISPERSAL

**Figure 1:**
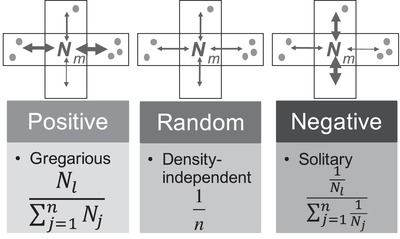
New models of density‐dependent dispersal implemented in the modified version of SPLATCHE2. The gray dots represent individuals in each neighbor deme. The width of the arrows denotes the amount of migrants sent from the central deme. The migration probability of individuals from deme *i* to deme *l* (*P_i,l_*) was considered to be either dependent (negative and positive models) or independent of neighbor densities (random model). The values of *j* and *n* represent one of the four available neighbor demes, respectively.

Organisms may disperse because they are attracted by conspecifics, or conversely because they prefer depopulated areas. We modified SPLATCHE2 by implementing two models of dispersal to reflect those behaviors, referred to as positive or negative density‐dependent dispersal. For all dispersal models, the proportion of individuals emigrating from each deme at each generation is defined by the migration rate *m*. However, the direction of emigrants varies among models (Fig. 1). In the original version of SPLATCHE2, the migratory probabilities *P_i,l_* from deme *i* to *l* are density independent and are equal toward each of the *n* neighboring demes (a value of *P_i,l_* = 0.25 if 4 neighbors, random model in Fig. 1). This model was used by Currat et al. (2008) and represents our null hypothesis to test the effect of positive and negative density‐dependent dispersal. For those two last models, the migratory probability *P_i,l_* from a deme *i* to a deme *l* of the same species (grid) depends on the densities in *n* neighboring demes as shown in Figure 1. For the positive model $P_{i,l}=N_{l}/\sum_{j=1}^{n}N_{j}$, where *N_l_* represents the population size of a deme *l* that is contiguous to deme *i*, whereas *N_j_* denotes the density in each of the *n* demes contiguous to *i*. It simulates the more gregarious or social behavior of individuals attracted by conspecifics. The negative model is computed as: $P_{i,l}=\frac{1}{N_{l}}/\sum_{j=1}^{n}\frac{1}{N_{j}}$, characterizing the more solitary or territorial behavior of individuals avoiding conspecifics during spatial dispersal. In other words, the intensity of emigration from a focal deme *i* toward a target deme *l* is proportional to the density in the target deme relatively to the densities in all possible *n* destinations (maximum *n* = 4 as a two‐dimensional stepping‐stone framework is used). For instance, if there are five individuals in deme *l* and 10 individuals in the three other neighboring demes, the positive model will give a probability $P_{i,l}$ of migrating from *i* to *l* equal to 5/35 = 0.145 and a probability equal to 0.285 of migrating to each of the three other alternative destinations. The same numerical example for the negative model gives $P_{i,l}\;$= (1/5)/(5/10) = 0.4 and 0.2 toward each other destination, respectively. To avoid undefined values, we replaced zero abundance by 0.1. This also allows empty demes to have a (small) chance to be colonized in case of positive dispersal and a (small) amount of gene flow toward populated demes in case of negative dispersal. The probability for an emigrant to go to any direction is always between 0 and 1, depending on the respective densities in each direction, while the sum of probabilities toward all neighbors is always equal to 1.

### COMPARISON BETWEEN VARIOUS DISPERSAL MODELS IN A VIRTUAL SQUARE WORLD

In a first part, we reproduced the simulation scheme of Currat et al. (2008) but included density‐dependent dispersal to investigate how it affects their conclusions about asymmetrical introgression between both species (which was shown to be much higher for the invasive species). We thus used a two‐dimensional square space of 100 × 100 demes, in which an invasive species started a range expansion 1500 years ago from the center of the grid. At the onset of the expansion, the area is already occupied by a local species, which can hybridize with the invasive one at various rates regulated by the parameter *γ*. The invasive species was introduced with 50 individuals in the center of the area and colonizes it in a similar way as implemented by Currat et al. (2008), which used the center to avoid “border effects” (i.e., emigrants unable to migrate in one or two directions due to the border of the square world). For each demographic scenario, we then performed 10,000 backward coalescent simulations representing 10,000 independent neutral genetic loci. The use of the coalescence algorithm implemented in SPLATCHE2 allows an enormous gain in computational efficiency because it only requires simulating the sampled genes and their ancestral lineages to represent the entire population. To assess the final proportion of introgressed genes in each species, we sampled 40 gene copies in 25 equally spaced demes, summing up to 1000 sampled genes for each locus. We did not use molecular information to estimate the amount of introgression but traced the genealogy back and computed the proportion of lineages sampled at present time in species *i* which was in species *j* at the onset of the expansion (i.e., lineages introgressed from one species to the other), following the procedure described in details in Currat et al. (2008). We then averaged this proportion over the 10,000 independent loci.

We explored various scenarios assessing the effect of different carrying capacities (*K*) and number of migrants among neighbor demes (*Km*). We present five scenarios without competition between species (NC, Table 1), whereas seven additional scenarios with competition (C) are presented in supplementary material (Table S1). Without competition (scenarios NC), both species are supposed to use different niches and can therefore coexist and interbreed until the end of our simulations. We decided to present those NC scenarios in the main text because introgression can be computed in both local and invasive organisms. In scenarios C, the invasive species has a competitive advantage due to larger carrying capacity, driving local species to extinction. In this last case, interbreeding occurred exclusively at the edge of the wave of expansion, in which both species coexist for a duration depending on the combination of parameters. All scenarios NC and C are identical to those of Currat *et al* (2008), except the mode of dispersal (Fig. 1), and are used for a direct comparison among models.

**Table 1:** Simulated scenarios with parameter values

	Local species	Invasive species		
Scenario	*K*	*Km*	*K*	*Km*
NC1	50	10	50	10
NC2	500	10	500	10
NC3	500	100	500	100
NC4	50	10	500	100
NC5	500	100	50	10

They represent the expansion of invasive organisms in an area occupied by a local species without interspecific competition (Currat et al. 2008). *K* is the carrying capacity and *Km* is the number of emigrants sent to neighbor demes when carrying capacity is reached. The intrinsic growth rate (*r*) is fixed to 0.5 in all scenarios. Scenarios incorporating interspecific competition are shown in Table S1.

### CASE STUDY: APPLICATION TO EUROPEAN WILDCATS IN THE JURA AREA

In a second part, we applied our approach to the case of introgression between wildcats and domestic cats in the Jura Mountains (Switzerland). Under a spatially explicit simulation framework using SPLATCHE2, Nussberger et al. (2018) were able to detect a recent range expansion of European wildcats in this area over the last 50 years, despite the fact that wildcats were considered to be almost extinct in 1962 when there was a change in protection policy. We thus extended the simulation framework used in Nussberger et al. (2018) to investigate if the incorporation of density‐dependent dispersal may improves the accuracy of the model of wildcat expansion with hybridization to explain the observed evidence of introgression. Note that in this case, the expanding population is the indigenous one (wildcats) recovering its past habitat.

The framework of Nussberger et al. (2018) was designed to study the specific case of interactions between wild and domestic cats in the Swiss Jura area using a random dispersal mode. We reproduced this simulation framework but included positive and negative dispersal model in addition to the random one. Note that we only reproduced the best estimated scenario from Nussberger et al (2018), which represents a wildcat re‐expansion in a habitat occupied by domestic cats. We simulated an array of 256 demes of 25 km^2^ each, roughly representing the Swiss Jura region (∼6400 km^2^). In this landscape, 16 demes represent habitat exclusively suitable for wildcats, 48 demes are potentially shared by both wildcats and domestic cats, while the remaining 192 demes are exclusively used by domestic cats. About 1600 km^2^ are suitable for wildcats. We assumed domestic cat omnipresence in the surroundings of a small core region with exclusively wildcat presence.

The values of demographic parameters and observed introgression (Table 2) are based on Nussberger et al. (2018). As in Nussberger et al. (2018), we used variable K among demes by drawing values around the mean given in Table 2. We used as genetic information the proportion of introgressed loci on autosomes, Y chromosome, and mitochondria, which have been estimated from 68 autosomal nuclear, four mtDNA, and two Y‐chromosome SNP‐markers (Nussberger et al. 2018). All SNPs were chosen to be highly differentiated between the two cat species (Nussberger et al. 2013). We consider a single genetic population of wildcats found in the Jura on both side of the political border between France and Switzerland (Table 2).

**Table 2:** Observed genetic introgression and parameter values used in the case study of hybridization between European wildcats and domestic cats in the Jura region (see Nussberger et al. 2018)

	Wildcat	Domestic cat
Genetic introgression		
Autosomal	7–18%	0–5%
mtDNA	9–22%	0–3%
Y‐chromosome	0–9%	0%
Model parameters		
Generation time (years)	3	3
Interbreeding success rate (γ)	0–0.4	0–0.4
Growth rate (*r*)	1.0	1.0
Migration rate (*m*)	0.18	0.18
Carrying capacity (*K*)	12	70

We assessed the probability of explaining the observed introgression levels between both cats for a range of interbreeding success rate parameter (*γ*) in each model of spatial dispersal (positive, negative, or random). This computation was done independently for all three marker‐categories (autosomal, mitochondrial, and Y‐chromosome). We computed a dummy variable evaluating whether simulated levels of introgression fell within (coded 1) or outside (coded 0) the 95% confidence interval around the observed values (Table 2). We used this variable as the response in a generalized additive model (GAM) with binomial error. A GAM is a regression method that allows incorporating the nonlinear effect of the *γ* parameter, which is used as explanatory variable (Hastie and Tibshirani 1986). The response variable is therefore *P*(1*|γ*), which is the probability of the value of *γ* given that the simulated introgression falls within the 95% CI. The dispersal models (random, positive, and negative) were incorporated as a three‐level factor variable and further analyzed by means of a post hoc pairwise analysis with a Bonferroni correction applied to the regression coefficients. These analyses were performed by using the software *R* (R Development Core Team 2017). The packages *mgcv* (Wood 2006) and *multcomp* (Hothorn et al. 2008) were used to implement the GAMs and the pairwise comparison among spatial dispersal modes, respectively. The accuracy of this method to assess parameter values is explored in Fig. S1.

## Results

### COMPARISON BETWEEN VARIOUS DISPERSAL MODELS IN A VIRTUAL SQUARE WORLD

#### Speed of range expansion of a single species

**Figure 2:**
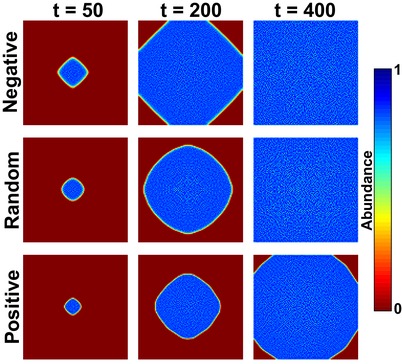
Range expansion of a theoretical species with different models of dispersal. The square space is composed of 10,000 demes. Population density in each deme is logistically regulated with a carrying capacity of 50 individuals (*K*). The abundance is represented as a proportion of the carrying capacity. The intrinsic growth rate (*r*) is fixed to 0.5 (scenario NC1, Table 1). The corresponding time is shown at the top of each column (*t* = number of generations). All expansion starts at *t* = 0 in the center deme.

We first modeled a single species invading a large unoccupied area of 10,000 demes (Fig. 2). The mode of density‐dependent dispersal influences the speed of the range expansion. Organisms that tend to avoid areas already occupied by conspecifics (negative model) experience a faster expansion, whereas organisms attracted by conspecifics (positive model) are slower to colonize the whole area when compared with density‐independent dispersal (random model).

#### Genetic introgression

**Figure 3:**
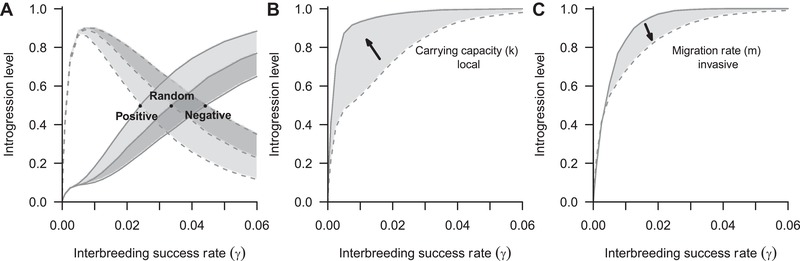
Effect of interbreeding success rate on the proportion of introgressed genes in a theoretical invasive and local species taken from the square world described in Figure 2. (A) Different models of spatial dispersal are presented for both local (dotted lines) and invasive (solid line) organisms (scenario NC4, Table 1); (B) effect of increasing local carrying capacity on the introgression of the invasive species, comparison between scenario NC5 (solid line) and NC1 (dotted line); (C) effect of decreasing migration rate of the invasive organisms on its introgression level, comparison between scenarios NC3 (solid line) and NC2 (dotted line). At the onset of the invasive expansion, the local species occupied the whole area and then interact with the invasive during 1,500 years. Introgression values are average over 10,000 stochastic simulations.

Once the species has colonized the whole area, we let it evolve until generation 1,000 and it is thereafter considered as the local species. This is the time required by the species to colonize the whole area and to reach demographic equilibrium for all explored scenarios. Then, a second species, hereafter called invasive, is introduced from the center of the grid. The invasive species may hybridize with the local one at various rates regulated by the value of *γ* (Fig. 3). Both species coexist and admix in the absence of interspecific competition. For all three models of spatial dispersal (i.e., positive, negative and random migratory response to conspecifics), we found that reciprocal introgression occurs at very low levels of interbreeding, but introgression starts to be asymmetrical toward the invasive species with higher *γ* values. A large introgression of local genes in the invasive species is obtained at relatively low *γ* (>5%) for all dispersal modes due to the demographic dynamics (Fig. 3A and Fig. S2). Introgression level in the invasive species is positively correlated with the local species density (Fig. 3B) but negatively correlated with its migration rate (Fig. 3C). Introgression of invasive genes in the local species follows the opposite trend (Fig. 3A). At very low frequency of interbreeding (*γ*), introgression of genes is found to be larger in the species with the smaller density (NC4 and NC5, Fig. S2), even when it is the local one (NC4, see Fig. 3A). Indeed, when the frequency of interbreeding is very low, it almost never happens during the spatial expansion of the invasive species, and mostly, if not only, at demographic equilibrium. The respective levels of introgression in both species thus only depend on their relative carrying capacities. However, as soon as the probability of interbreeding exceeds 5%, all scenarios result in a larger introgression of local genes in the invasive species and smaller introgression into the local organisms (see Fig. S2). The high introgression of neutral genes in the invasive organisms is thus dependent on a value of *γ* big enough to result in interbreeding events occurring at the front of the range expansion wave.

Although the positive and negative models of spatial dispersal exhibit the same general trend as the random model, they show some quantitative differences. To get equal introgression levels in both species, a lower interbreeding rate (*γ*) is required for the positive model as compared with the random model, and higher for the negative model (Fig. 3A). For instance, scenario NC4, in which the invasive species is much more abundant than the local one, requires around 4% of interbreeding to start having more introgression in the invasive species than in the local one for the random model, while it needs around 3% of interbreeding for the positive and 5% for the negative. In other words, when invasive individuals are attracted by conspecifics (positive model), this leads to more introgression in the invasive species and less in the local one, by comparison to random dispersal, whereas the opposite occurs when invasive individuals are repulsed by conspecifics (negative model). To depict this result, we computed the differences in the level of genetic introgression between both models of density‐dependent dispersal and the random model (Fig. S3). All models are equivalent at low frequency of interbreeding. The differences reach a maximum between 2% and 4% of *γ* . Higher interbreeding success rates tend to homogenize the results of all three models due to the near complete introgression of genes from the local to the invasive species. Scenarios with higher local densities (NC2, NC5, and NC3) show the smallest differences between both density‐dependent models and the random model. At a given frequency of interbreeding (*γ*), with the positive model, the invasive species is more introgressed and the local one is less introgressed than expected with the random model (Fig. S3A and S3B). However, the negative model exhibits the opposite trend, where the local species is much more introgressed and the invasive species is less introgressed than expected with the random model (Fig. S3C and S3D).

Similar results were found when interspecific competition is incorporated in the simulations (see Fig. S4). In these simulations, the invasive species progressively replaces the local one due to competition for resources and higher carrying capacity. Even if the local species is demographically extinct at the end of a simulation, its genes can still be found in the genome of invasive organisms when the interbreeding rate is large enough. Here again, the density‐dependent models of spatial dispersal (positive and negative) show the same general trend as the random model, with the same patterns of differences as described above for the noncompetition scenarios.

### CASE STUDY: APPLICATION TO EUROPEAN WILDCATS IN THE JURA AREA

We applied the three different models of spatial dispersal to a real case of hybridization between European wildcats and domestic cats in the Jura region. High introgression of genes from domestic cat is found in European wildcats in this region, whereas almost no introgression of wildcat genes is found in domestic cats (Table 2). Wildcats were considered to be nearly extinct, but the implementation of new policies for conservation helped to increase the number of individuals. A recent range expansion of wildcats during the last 50 years (∼17 generations) may have influenced hybridization with domestic cats. This scenario is similar to our previous theoretical scenario NC5, in which the local organisms (i.e., domestic cats) have higher carrying capacity (*K*) than the invasive individuals (i.e., wildcats).

**Figure 4:**
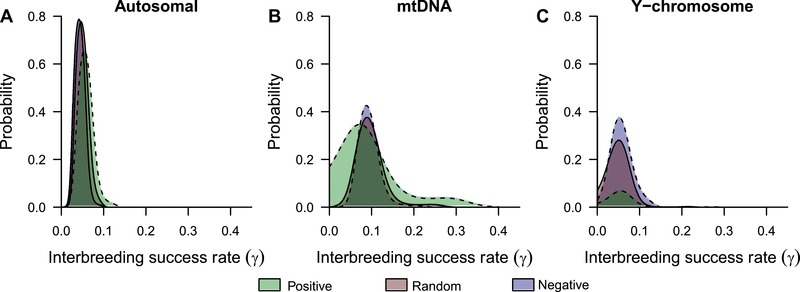
Probability of the three models of spatial dispersal in explaining the observed introgression between European wildcats and domestic cats. Various interbreeding success rate applied to three kinds of genetic markers are presented. The dotted line represents significant mean differences when comparing the density‐dependent models of spatial dispersal (negative and positive) with the density‐independent one (random). Bonferroni correction for multiple comparisons is considered (see Methods section).

Our results show that the negative density‐dependent model of spatial dispersal is consistently the most likely explanation for the current introgression of genes between both cats across all genetic markers (Fig. 4). The probability of simulations to explain field observations increases by 13% for autosomal markers (Fig. 4A), 10% for mtDNA (Fig. 4B), and 31% for the Y‐chromosome (Fig. 4C) when compared to the model with positive density‐dependent dispersal (less likely model). The difference is not significant between the random and negative model for the autosomal marker (*P* = 0.79), but it is significant for mtDNA and the Y chromosome (*P* values < 0.001). Moreover, the positive model is significantly less likely when compared with the random and with the negative model for all genetic markers (all *P* values < 0.001).

The most likely values of interbreeding success rate (*γ*) range between 4% and 9% considering all markers and models of spatial dispersal. Those values explain much more observed introgression when applied to the autosomal markers as compared to the mtDNA and Y‐chromosome markers (Fig. 4). An interbreeding rate of around 5% explains a maximum of 80% of introgression for the autosomal markers when using the negative model (Fig. 4A), and a minimum of 6% on the Y‐chromosome when using the positive model (Fig. 4C). For mtDNA, a higher value of interbreeding success rate is needed to explain a maximum of introgression, a *γ* around 7% for the positive and 9% for the random and negative models is needed to explain 30% and 40% of the introgression between both cats, respectively (Fig. 4B). Overall, these results show that the amount of explained introgression depends on the genetic marker analyzed, with more events of interbreeding needed to explain observed maternally inherited genes.

## Discussion

### DENSITY DEPENDENT DISPERSAL AND RANGE EXPANSION

Dispersal is a crucial process for both the successful colonization for species naturally expanding their range or for those settling new areas in response to human activities (Wilson et al. 2009). It is important to have accurate modeling tools to predict when and how an invasion might succeed, as attempts to reverse or control the negative effects of human‐translocated species on local biodiversity can be extremely costly and difficult (Hauser and McCarthy 2009). The introduction of exotic species may become ecologically detrimental for native taxa, either due to direct predation, competition or modification of habitat, but also due to the genetic impact of hybridization (Hall et al. 2006). Hybridization has been shown to have the potential to facilitate biological invasions and result in the decline of native genotype or the extinction of one or both parental species (Quilodrán et al. 2015, 2018a, 2018b).

The use of social signals during dispersal is widely documented in animals, for example in territorial or colonial birds (Kivelä et al. 2014), mammals (McGuire et al. 2009), reptiles (Spiegel et al. 2015), amphibians (González‐Bernal et al. 2014), fish (Bett and Hinch 2015), and even invertebrates (Donahue 2006). The presence of conspecifics can play a positive, negative or neutral effect when individuals are seeking habitat for survival or reproduction (Muller 1998). Here, we highlight the critical importance of incorporating behavioral responses toward conspecifics when projecting the dynamics of ecological and genetic interactions during range expansion.

Improving the predictive power of range expansion models, as we propose here by incorporating density‐dependent dispersal, is crucial to better understand how species respond to current and rapid environmental changes (Ponchon et al. 2015). In fact, traits involved in dispersal could exhibit adaptation during a range shift induced by climate change, in which individuals can escape locally degraded environments and colonize new suitable areas (Travis et al. 2013). In that case, new biodiversity can be produced either because populations become allopatrically structured or by making contact with sister species. There is strong evidence of extreme weather changes in recent years, namely on patterns of heat waves and precipitation, that have been linked to human influences (Coumou and Rahmstorf 2012). New ecological and genetic interactions are thus likely to be produced in the near future. The zone of hybridization can move in space and time when influenced by a changing climate, but the evolutionary consequences on local biodiversity are uncertain (Buggs 2007). Hybridization is also an evolutionary force that has been acting in the speciation process of numerous taxa (Brumfield 2010). However, it has also been presented as a mechanism for “speciation reversal” when breaking the independent evolution of recently separated groups (Vonlanthen et al. 2012).

### LESSONS FROM SIMULATIONS IN A VIRTUAL SQUARE WORLD

#### Asymmetrical introgression is the general expectation for neutral genes

Previous studies have highlighted the importance of density‐dependent dispersal in ecology and evolution, but they did not consider genetic aspects (e.g., Altwegg et al. 2013; Bocedi et al. 2014; Ponchon et al. 2015). Our work is thus the first to study the genetic dimension of density‐dependent dispersal during range expansion of an invasive species. For this, we developed two new models of density‐dependent dispersal during range expansion to study their influence on genetic introgression between local and invasive species when hybridization between them is possible. Here, we computed introgression levels with and without competition for environmental resources using the same demographic scenarios as in Currat et al. (2008). Our results show that their main conclusion of a larger introgression of neutral genes in the invasive species compared to the one in the local species, even when the level of admixture is very low, remains valid with both models of density‐dependent dispersal. Our results are quantitatively closer to the ones presented by Excoffier et al. (2014) as compared to those of Currat et al. (2008) because we used the same admixture model as Excoffier et al. (2014), which is better adapted to hybridization. Our two density‐dependent models present qualitatively similar trends, but a greater frequency of interbreeding (*γ*) is needed under negative and a smaller one under positive density‐dependent dispersal to get results similar to those obtained with random density‐independent dispersal.

This general pattern of asymmetric introgression between a native and an invasive species undergoing range expansion thus represents a null expectation for neutral genetic markers. Deviations from this neutral pattern are possible under the effect of selective pressures (e.g., Whitney et al. 2006; Chhatre et al. 2018). In addition, neutral polymorphisms may become advantageous in a new genetic or environmental background (e.g., Montoya‐Burgos 2011), making the outcomes more difficult to predict. Sex‐biased gene flow due, for instance, to a behavioral response to new phenotypic traits producing asymmetrical mating preferences (Meyer et al. 2006; While et al. 2015), or due to sex‐biased survival of hybrids (Bundus et al. 2015), may also disturb this null expectation.

#### Density‐dependent dispersal influences demographic and introgression patterns

We show that negative or positive density‐dependent response toward conspecifics affects both the colonization speed and the pattern of introgression between interacting species. Altwegg et al. (2013) found a qualitatively similar result in terms of colonization time, in which colonization of a given area occurs much faster for negative density‐dependent emigration than for positive. In addition, densities of the invasive species are lower at the wave front for positive and higher for negative dispersal.

The quantity of introgression also differs when comparing both models to one completely independent of densities (random dispersal). For a given value of interbreeding success rate (*γ*), the amount of introgression in the invasive species is lower under negative and higher under positive density‐dependent dispersal. This result is influenced by the slower colonization time resulting from the positive model, which lengthens the overall cohabitation period and maximizes the effect of the wave front to amplify introgression (Currat et al. 2008). In addition, the low‐density populations at the wave front receive fewer migrants from behind under the positive model, which is favorable for introgression to occur (Petit and Excoffier 2009). The opposite trend is observed in the local species, with larger introgression under negative density‐dependent dispersal and lower under positive density‐dependent dispersal. This is influenced by the faster range expansion under negative density‐dependent dispersal and the demographic imbalance between both species, in which the rarest species is more likely to hybridize with a more abundant one (Hubbs 1955). The local species is in demographic equilibrium, thus the invasive one, escaping their conspecifics under negative density‐dependent dispersal, have a probability to find a heterospecific partner during the breeding period (McCracken and Wilson 2011). However, heterospecific mating is compensated by the higher number of conspecific migrants from the core of expansion, which tend to move attracted by the lower densities. Introgression in the invasive species at the wave front is therefore limited by the larger gene flow coming from the core (Petit and Excoffier 2009). Consequently, invasive populations are growing quickly and the probability for the local organisms to receive invader genes by hybridization also increases. Our series of simulations highlight the usefulness of computer simulations to study complex systems, such as exploring the genetic outcomes resulting from interacting processes, namely migration, demographic growth, and hybridization.

### LESSONS FROM OUR CASE STUDY

#### Negative density‐dependent dispersal explains better introgression in European Wildcats

Wildcats were considered to be almost extinct in the Swiss Jura, but the protection policy changes and the new legislation allowed significant recovery of the population during the last 50 years (Nussberger et al. 2014). They are still considered a threatened species in several countries in Europe, with the primary risk being hybridization with domestic cats (Yamaguchi et al. 2015). We thus applied the three models of spatial dispersal to the case of hybridization between European wildcats and domestic cats under a simulation framework specifically designed for this situation (Nussberger et al. 2018). The goal was to evaluate which model best explained the observed introgression.

Our results showed that in accordance with the expectation, the negative dispersal model was consistently the most likely one to explain the observed hybridization in nature. It is interesting to note that even within the short time frame of our simulations (17 generations, ∼50 years), we are able to reject the positive model of density‐dependent dispersal and to improve the goodness of fit by 30% using the negative model. Allowing more time for ecological and genetic interactions may probably increase the power to differentiate among models of spatial dispersal.

Wildcats are solitary individuals and their home ranges rarely overlap, almost never for females (Biró et al. 2004). They associate exclusively for mating (Sunquist and Sunquist 2002), and may thus disperse to avoid agonistic encounters due to territorial behavior between individuals (Corbett 1979). Our negative model is more accurate for this type of behavior, in which territorial individuals avoid conspecifics during dispersal, increasing the probability of mating with a heterospecific during the breeding period—in this case domestic cats. Domestic cats can either live alone, in groups, or a mixture of this life style (Corbett 1979). They are distributed worldwide, associated with humans as pets or for controlling agricultural pests (Turner 2000). Domestic cats were brought into Europe already by the Romans (Faure and Kitchener 2009). Thus, domestic cats, including feral cats, were already present when wildcats started the recolonization in the 20th century.

The interbreeding success rate (*γ*) that best explains observed introgression is estimated around 5% for the autosomal and Y‐chromosome markers. It means that about 5% of contacts between wildcats and domestic cats result in the birth of hybrid offspring. Higher values are observed for mtDNA (∼9%). Male wildcats display more explorative spatial behavior when looking for a partner than females (Daniels et al. 2001). They are therefore more likely to be involved in heterospecific mating with domestic females, resulting in hybrids carrying domestic mtDNA. This behavior contributes to explain the observed higher introgression and the higher interbreeding rate in mitochondrial genes compared to the Y‐chromosome. The more exploratory behavior of male wildcats compared to females (Liberg and Sandell 1988; Daniels et al. 2001; Devillard et al. 2004; Beugin et al. 2016) may also explain why the Y‐chromosome is more sensitive than autosomes or mtDNA to the model of density‐dependent dispersal (the accuracy of the models varying among models by 13% for autosomal markers, 10% for mtDNA, and 31% for the Y‐chromosome). Indeed, we can consider that the behavior of males is more different from the random model than the behavior of females, and thus easier to differentiate with our modeling approach. Moreover, the genetic introgression observed in the male line (Y‐chromosome) is quite similar in the wildcat and domestic cats, and is rather low. In comparison, females have more pronounced asymmetry, in which the female line of wildcats is rather highly introgressed, whereas domestic females do not show much introgression. The less asymmetry shown in the male line (Y‐chromosome, Table 2) may be better explained by the negative dispersal than by the two alternative models.

The pattern of asymmetrical and larger introgression in wildcats than in domestic cats may seem counterintuitive given that the Africa derived domestic cats are invasive in Europe. Indeed, the null expectation presented by Currat et al. (2008) and confirmed by our simulations anticipated higher introgression in the invasive species. However, the invasive is not always nonindigenous and the local is not necessarily a native taxon. In this case, it is a recolonization of lost territory by wildcat at the expense of the nonindigenous domestic cats. This makes the wildcats the invasive organisms in the context modeled here.

The recent range expansion of this threatened species represents a success for conservation management. However, the policy should still be amended to avoid hybridization by controlling both feral and domestic animals in the area suitable for colonization by wildcats—at least as long as potentially negative effects of hybridization cannot be excluded (Witzenberger and Hochkirch 2014).

## Conclusion

A large introgression from a local species into a species invading its range is expected for neutral genes, whatever the mode of spatial dispersal. Our models of spatial dispersal differ quantitatively in terms of colonization time and level of introgression between local and invasive species, but the general trend first observed by Currat et al. (2008) is confirmed by all our simulations. However, the quantitative differences highlighted here may be significant when one wants to study the genetic consequences of range expansions on specific organisms, as illustrated with the higher accuracy of the most adequate model of spatial dispersal in our case study on European wildcats. Including density‐dependent dispersal, as we did here, may improve the predictive power of models of range expansion. Our spatially explicit approach constitutes a valuable tool in evolutionary and conservation biology that can be used in a variety of biological issues, including the set‐up of management recommendations for threatened species and the study of past and future evolution of interacting taxa.

Associate Editor: S. M. Flaxman

Handling Editor: M. Servedio

## Supporting information


**Figure S1**. Accuracy of the method to estimate the interbreeding success rate (*γ*).
**Figure S2**. Effect of interbreeding success rate on the proportion of introgressed genes in a theoretical invasive and local species taken from the square world described in Fig. 2 of the main text.
**Figure S3**. Differences in the proportion of introgressed genes of positive and negative model compare to the random model of spatial dispersal.
**Table S1**. Parameter values for seven scenarios of an invasive species in range expansion in an area occupied by a local species with interspecific competition.
**Figure S4**. Effect of interbreeding success rate on the proportion of introgressed genes in a theoretical invasive species taken from the square world described in Fig. 2 of the main text.
**Figure S5**. Representation of the spatial scenario of our case study about European wildcats and domestic cats (modified from Nussberger et al. 2018).

## References

[evo13704-bib-0001] Aguillon, S. M. , and R. A. Duckworth . 2015 Kin aggression and resource availability influence phenotype‐dependent dispersal in a passerine bird. Behav. Ecol. Sociobiol. 69:625–633.

[evo13704-bib-0002] Altwegg, R. , Y. C. Collingham , B. Erni , and B. Huntley . 2013 Density‐dependent dispersal and the speed of range expansions. Divers. Distrib. 19:60–68.

[evo13704-bib-0003] Arnold, M. L. , and N. H. Martin . 2010 Hybrid fitness across time and habitats. Trends Ecol. Evol. 25:530–536. 20598770 10.1016/j.tree.2010.06.005

[evo13704-bib-0004] Bett, N. N. , and S. G. Hinch . 2015 Attraction of migrating adult sockeye salmon to conspecifics in the absence of natal chemical cues. Behav. Ecol. 26:1180–1187.

[evo13704-bib-0005] Beugin, M.‐P. , G. Leblanc , G. Queney , E. Natoli , and D. Pontier . 2016 Female in the inside, male in the outside: insights into the spatial organization of a European wildcat population. Conserv. Genet. 17:1405–1415.

[evo13704-bib-0006] Biró, Z. , L. Szemethy , and M. Heltai . 2004 Home range sizes of wildcats (*Felis silvestris*) and feral domestic cats (*Felis silvestris* f. catus) in a hilly region of Hungary. Mammalian Biology 69:302–310.

[evo13704-bib-0007] Bocedi, G. , D. Zurell , B. Reineking , and J. M. Travis . 2014 Mechanistic modelling of animal dispersal offers new insights into range expansion dynamics across fragmented landscapes. Ecography 37:1240–1253.

[evo13704-bib-0008] Brown, R. M. , R. A. Nichols , C. G. Faulkes , C. G. Jones , L. Bugoni , V. Tatayah , D. Gottelli , and W. C. Jordan . 2010 Range expansion and hybridization in Round Island petrels (Pterodroma spp.): evidence from microsatellite genotypes. Mol. Ecol. 19:3157–3170. 20618891 10.1111/j.1365-294X.2010.04719.x

[evo13704-bib-0009] Brumfield, R. T. 2010 Speciation genetics of biological invasions with hybridization. Mol. Ecol. 19:5079–5083. 21091659 10.1111/j.1365-294X.2010.04896.x

[evo13704-bib-0010] Buggs, R. J. A. 2007 Empirical study of hybrid zone movement. Heredity 99:301–312. 17611495 10.1038/sj.hdy.6800997

[evo13704-bib-0011] Bundus, J. D. , R. Alaei , and A. D. Cutter . 2015 Gametic selection, developmental trajectories, and extrinsic heterogeneity in Haldane's rule. Evolution 69:2005–2017. 26102479 10.1111/evo.12708

[evo13704-bib-0012] Chhatre, V. E. , L. M. Evans , S. P. DiFazio , and S. R. Keller . 2018 Adaptive introgression and maintenance of a trispecies hybrid complex in range‐edge populations of Populus. Mol. Ecol. 27:4820–4838. 30071141 10.1111/mec.14820

[evo13704-bib-0013] Corbett, L. K. 1979 Feeding ecology and social organization of wildcats (*Felis silvestris*) and domestic cats (*Felis catus*) in Scotland. PhD diss. University of Aberdeen, Aberdeen, UK.

[evo13704-bib-0014] Coumou, D. , and S. Rahmstorf . 2012 A decade of weather extremes. Nat. Clim. Chang. 2:491–496.

[evo13704-bib-0015] Currat, M. , N. Ray , and L. Excoffier . 2004 SPLATCHE: a program to simulate genetic diversity taking into account environmental heterogeneity. Mol. Ecol. Notes 4:139–142.

[evo13704-bib-0016] Currat, M. , M. Ruedi , R. J. Petit , and L. Excoffier . 2008 The hidden side of invasions: massive introgression by local genes. Evolution 62:1908–1920. 18452573 10.1111/j.1558-5646.2008.00413.x

[evo13704-bib-0017] Daniels, M. J. , M. A. Beaumont , P. J. Johnson , D. Balharry , D. W. Macdonald , and E. Barratt . 2001 Ecology and genetics of wild‐living cats in the north‐east of Scotland and the implications for the conservation of the wildcat. J. Appl. Ecol. 38:146–161.

[evo13704-bib-0018] Devillard, S. , E. J. Say , and D. Pontier . 2004 Molecular and behavioural analyses reveal male‐biased dispersal between social groups of domestic cats. Ecoscience 11:175–180.

[evo13704-bib-0019] Donahue, M. J. 2006 Allee effects and conspecific cueing jointly lead to conspecific attraction. Oecologia 149:33–43. 16688470 10.1007/s00442-006-0419-y

[evo13704-bib-0020] Dötterer, M. , and F. Bernhart . 1996 The occurrence of wildcats in the southern Swiss Jura Mountains. Acta Theriol. 41:205–210.

[evo13704-bib-0021] Duelli, P. , and D. Agosti . 1994 Rote Listen der gefährdeten Tierarten der Schweiz. BUWAL, Bundesamt für Umwelt, Wald und Landschaft.

[evo13704-bib-0022] Excoffier, L. , C. S. Quilodrán , and M. Currat . 2014 Models of hybridization during range expansions and their application to recent human evolution Pp. 122–137 *in* Derevianko A. and Shunkov M., eds. Cultural developments in the Eurasian Paleolithic and the origin of anatomically modern humans. Department of the Institute of Archaeology and Ethnography SB RAS, Novosibirsk, Russia.

[evo13704-bib-0023] Faure, E. , and A. C. Kitchener . 2009 An archaeological and historical review of the relationships between felids and people. Anthrozoös 22:221–238.

[evo13704-bib-0024] Fretwell, S. D. , and H. L. Lucas . 1969 On territorial behavior and other factors influencing habitat distribution in bird. Acta Biotheor. 19:16–36.

[evo13704-bib-0025] Garcia, M. G. , R. S. Silva , M. A. Carniello , J. W. Veldman , A. A. B. Rossi , and L. O. de Oliveira . 2011 Molecular evidence of cryptic speciation, historical range expansion, and recent intraspecific hybridization in the neotropical seasonal forest tree *Cedrela fissilis* (Meliaceae). Mol. Phylogenet. Evol. 61:639–649. 21930224 10.1016/j.ympev.2011.08.026

[evo13704-bib-0026] Garcia‐Elfring, A. , R. Barrett , M. Combs , T. Davies , J. Munshi‐South , and V. Millien . 2017 Admixture on the northern front: population genomics of range expansion in the white‐footed mouse (*Peromyscus leucopus*) and secondary contact with the deer mouse (*Peromyscus maniculatus*). Heredity 119:447–458. 28902189 10.1038/hdy.2017.57 PMC5677999

[evo13704-bib-0027] González‐Bernal, E. , G. P. Brown , and R. Shine . 2014 Invasive cane toads: Social facilitation depends upon an individual's personality. PLoS One 9:e102880. 25033047 10.1371/journal.pone.0102880 PMC4102590

[evo13704-bib-0028] Hall, R. J. , A. Hastings , and D. R. Ayres . 2006 Explaining the explosion: modelling hybrid invasions. Proc. R. Soc. B‐Biol. Sci. 273:1385–1389. 10.1098/rspb.2006.3473 PMC1560304 16777727

[evo13704-bib-0029] Hastie, T. , and R. Tibshirani . 1986 Generalized additive models. Statist. Sci. 1:297–318. 10.1177/096228029500400302 8548102

[evo13704-bib-0030] Hauser, C. E. and M. A. McCarthy . 2009 Streamlining ‘search and destroy’: cost‐effective surveillance for invasive species management. Ecol. Lett. 12:683–692. 19453617 10.1111/j.1461-0248.2009.01323.x

[evo13704-bib-0031] Hothorn, T. , F. Bretz , and P. Westfall . 2008 Simultaneous inference in general parametric models. Biom. J. 50:346–363. 18481363 10.1002/bimj.200810425

[evo13704-bib-0032] Hubbs, C. L. 1955 Hybridization between fish species in nature. Syst. Zool. 4:1–20.

[evo13704-bib-0033] Johannesen, J. , B. Johannesen , E. Griebeler , I. Baran , M. Tunc , A. Kiefer , and M. Veith . 2006 Distortion of symmetrical introgression in a hybrid zone: evidence for locus‐specific selection and uni‐directional range expansion. J. Evol. Biol. 19:705–716. 16674567 10.1111/j.1420-9101.2005.01064.x

[evo13704-bib-0034] Kivelä, S. M. , J.‐T. Seppänen , O. Ovaskainen , B. Doligez , L. Gustafsson , M. Mönkkönen , and J. T. Forsman . 2014 The past and the present in decision‐making: the use of conspecific and heterospecific cues in nest site selection. Ecology 95:3428–3439.

[evo13704-bib-0035] Liberg, O. , and M. Sandell . 1988 Räumliche Organisation und Fortpflanzungsstrategien der domestizierten Hauskatze und anderer Feliden. Pp. 103–123 *in* Turner D. and Bateson P., eds. Die domestizierte Katze: Eine wissenschaftliche Betrachtung ihres Verhaltens. Albert Müller Verlag, Rueschlikon‐Zurich.

[evo13704-bib-0036] Lotka, A. J. 1932 The growth of mixed populations: two species competing for a common food supply. J. Wash. Acad. Sci. 22:461–469.

[evo13704-bib-0037] Mastrantonio, V. , D. Porretta , S. Urbanelli , G. Crasta , and G. Nascetti . 2016 Dynamics of mtDNA introgression during species range expansion: insights from an experimental longitudinal study. Sci. Rep. 6:30355. 27460445 10.1038/srep30355 PMC4962091

[evo13704-bib-0038] McCracken, K. G. , and R. E. Wilson . 2011 Gene flow and hybridization between numerically imbalanced populations of two duck species in the Falkland Islands. PLoS One 8:e82664. 10.1371/journal.pone.0023173 PMC3162561 21887236

[evo13704-bib-0039] McGuire, B. , M. K. Oli , and L. L. Getz . 2009 Effects of conspecific and heterospecific residents on patterns of immigration in two species of voles. Acta Theriol. 54:321–332.

[evo13704-bib-0040] Meyer, A. , W. Salzburger , and M. Schartl . 2006 Hybrid origin of a swordtail species (Teleostei: *Xiphophorus clemenciae*) driven by sexual selection. Mol. Ecol. 15:721–730. 16499697 10.1111/j.1365-294X.2006.02810.x

[evo13704-bib-0041] Montoya‐Burgos, J. I. 2011 Patterns of positive selection and neutral evolution in the protein‐coding genes of Tetraodon and Takifugu. PLoS One 6:e24800. 21935469 10.1371/journal.pone.0024800 PMC3172292

[evo13704-bib-0042] Muller, K. L. 1998 The role of conspecifics in habitat settlement in a territorial grasshopper. Anim. Behav. 56:479–485. 9787039 10.1006/anbe.1998.0806

[evo13704-bib-0043] Neiva, J. , G. A. Pearson , M. Valero , and E. A. Serrao . 2010 Surfing the wave on a borrowed board: range expansion and spread of introgressed organellar genomes in the seaweed *Fucus ceranoides* L. Mol. Ecol. 19:4812–4822. 20958817 10.1111/j.1365-294X.2010.04853.x

[evo13704-bib-0044] Nussberger, B. 2013 Assessing Introgression between European wildcats ( *Felis silvestris silvestris*) and Domestic cats ( *Felis silvestris catus*). Institute of Evolutionary Biology and Environmental Studies. University of Zurich, Zurich, Switzerland.

[evo13704-bib-0045] Nussberger, B. , D. Weber , B. Hefti‐Gautschi , and P. Lüps . 2007 Neuester Stand des Nachweises und der Verbreitung der Waldkatze (*Felis silvestris*) in der Schweiz. Mitteilungen der Naturforschenden Gesellschaft in Bern 64:67–80.

[evo13704-bib-0046] Nussberger, B. , M. P. Greminger , C. Grossen , L. F. Keller , and P. Wandeler . 2013 Development of SNP markers identifying European wildcats, domestic cats, and their admixed progeny. Mol. Ecol. Resour. 13:447–460. 23398610 10.1111/1755-0998.12075

[evo13704-bib-0047] Nussberger, B. , P. Wandeler , D. Weber , and L. Keller . 2014 Monitoring introgression in European wildcats in the Swiss Jura. Conserv. Genet. 15:1219–1230.

[evo13704-bib-0048] Nussberger, B. , M. Currat , C. Quilodran , N. Ponta , and L. Keller . 2018 Range expansion as an explanation for introgression in European wildcats. Biol. Conserv. 218:49–56.

[evo13704-bib-0049] Petit, R. J. , and L. Excoffier . 2009 Gene flow and species delimitation. Trends Ecol. Evol. 24:386–393. 19409650 10.1016/j.tree.2009.02.011

[evo13704-bib-0050] Ponchon, A. , R. Garnier , D. Grémillet , and T. Boulinier . 2015 Predicting population responses to environmental change: the importance of considering informed dispersal strategies in spatially structured population models. Divers. Distrib. 21:88–100.

[evo13704-bib-0051] Quilodrán, C. S. , M. Currat , and J. I. Montoya‐Burgos . 2014a A general model of distant hybridization reveals the conditions for extinction in Atlantic salmon and brown trout. PLoS One 9:e101736. 25003336 10.1371/journal.pone.0101736 PMC4086968

[evo13704-bib-0052] Quilodrán, C. S. , C. F. Estades , and R. A. Vásquez . 2014b Conspecific effect on habitat selection of a territorial cavity‐nesting bird. Wilson J. Ornithol. 126:534–543.

[evo13704-bib-0053] Quilodrán, C. S. , J. I. Montoya‐Burgos , and M. Currat . 2015 Modelling interspecific hybridization with genome exclusion to identify conservation actions: the case of native and invasive *Pelophylax* waterfrogs. Evol. Appl. 8:199–210. 25685194 10.1111/eva.12245 PMC4319866

[evo13704-bib-0054] Quilodrán, C. S. , F. Austerlitz , M. Currat , and J. I. Montoya‐Burgos . 2018a Cryptic biological invasions: a general model of hybridization. Sci. Rep. 8:2414. 29402926 10.1038/s41598-018-20543-6 PMC5799175

[evo13704-bib-0055] Quilodrán, C. S. , M. Currat , and J. I. Montoya‐Burgos . 2018b Effect of hybridization with genome exclusion on extinction risk. Conserv. Biol. 32:1139–1149. 29691912 10.1111/cobi.13120

[evo13704-bib-0056] R Development Core Team . 2017 R: a language and environment for statistical computing. R Foundation for Statistical Computing, Vienna, Austria.

[evo13704-bib-0057] Ray, N. , M. Currat , M. Foll , and L. Excoffier . 2010 SPLATCHE2: a spatially explicit simulation framework for complex demography, genetic admixture and recombination. Bioinformatics 26:2993–2994. 20956243 10.1093/bioinformatics/btq579

[evo13704-bib-0058] Ren, G. P. , R. J. Abbott , Y. F. Zhou , L. R. Zhang , Y. L. Peng , and J. Q. Liu . 2012 Genetic divergence, range expansion and possible homoploid hybrid speciation among pine species in Northeast China. Heredity 108:552–562. 22187083 10.1038/hdy.2011.123 PMC3330684

[evo13704-bib-0059] Rushing, C. S. , M. R. Dudash , and P. P. Marra . 2015 Habitat features and long‐distance dispersal modify the use of social information by a long‐distance migratory bird. J. Anim. Ecol. 84:1469–1479. 26061822 10.1111/1365-2656.12395

[evo13704-bib-0060] Say, L. , S. Devillard , F. Léger , D. Pontier , and S. Ruette . 2012 Distribution and spatial genetic structure of European wildcat in France. Anim. Conserv. 15:18–27.

[evo13704-bib-0061] Sommer, R. , and N. Benecke . 2006 Late Pleistocene and Holocene development of the felid fauna (Felidae) of Europe: a review. J. Zool. 269:7–19.

[evo13704-bib-0062] Spiegel, O. , S. T. Leu , A. Sih , S. S. Godfrey , and C. M. Bull . 2015 When the going gets tough: behavioural type‐dependent space use in the sleepy lizard changes as the season dries. Proc. R. Soc. B 282 10.1098/rspb.2015.1768 PMC4685807 26609082

[evo13704-bib-0063] Stahl, P. , and M. Artois . 1994 Status and conservation of the wildcat (*Felis silvestris*) in Europe and around the Mediterranean rim. Council of Europe, Strasbourg, France.

[evo13704-bib-0064] Sunquist, M. , and F. Sunquist . 2002 Wild cats of the world. Univ. of Chicago Press, Chicago, IL.

[evo13704-bib-0065] Szostek, K. L. , M. Schaub , and P. H. Becker . 2014 Immigrants are attracted by local pre‐breeders and recruits in a seabird colony. J. Anim. Ecol. 83:1015–1024. 24460741 10.1111/1365-2656.12206

[evo13704-bib-0066] Travis, J. M. , M. Delgado , G. Bocedi , M. Baguette , K. Bartoń , D. Bonte , I. Boulangeat , J. A. Hodgson , A. Kubisch , and V. Penteriani . 2013 Dispersal and species’ responses to climate change. Oikos 122:1532–1540.

[evo13704-bib-0067] Turner, D. C. 2000 The domestic cat: the biology of its behaviour. Cambridge Univ. Press, Cambridge, U.K.

[evo13704-bib-0068] Volterra, V. 1928 Variations and fluctuations of the number of individuals in animal species living together. J. Cons. Int. Explor. Mer 3:3–51.

[evo13704-bib-0069] Vonlanthen, P. , D. Bittner , A. G. Hudson , K. A. Young , R. Muller , B. Lundsgaard‐Hansen , D. Roy , S. Di Piazza , C. R. Largiader , and O. Seehausen . 2012 Eutrophication causes speciation reversal in whitefish adaptive radiations. Nature 482:357–362. 22337055 10.1038/nature10824

[evo13704-bib-0070] While, G. M. , S. Michaelides , R. J. Heathcote , H. E. MacGregor , N. Zajac , J. Beninde , P. Carazo , G. Pérez i de Lanuza , R. Sacchi , and M. A. Zuffi . 2015 Sexual selection drives asymmetric introgression in wall lizards. Ecol. Lett. 18:1366–1375. 26468006 10.1111/ele.12531

[evo13704-bib-0071] Whitney, K. D. , R. A. Randell , and L. H. Rieseberg . 2006 Adaptive introgression of herbivore resistance traits in the weedy sunflower *Helianthus annuus* . Am. Nat. 167:794–807. 16649157 10.1086/504606

[evo13704-bib-0072] Wilson, J. R. , E. E. Dormontt , P. J. Prentis , A. J. Lowe , and D. M. Richardson . 2009 Something in the way you move: dispersal pathways affect invasion success. Trends Ecol. Evol. 24:136–144. 19178981 10.1016/j.tree.2008.10.007

[evo13704-bib-0073] Witzenberger, K. A. , and A. Hochkirch . 2014 The genetic integrity of the ex situ population of the European wildcat (*Felis silvestris silvestris*) is seriously threatened by introgression from domestic cats (*Felis silvestris catus*). PLoS One 9:e106083. 25162450 10.1371/journal.pone.0106083 PMC4146591

[evo13704-bib-0074] Wood, S. 2006 Generalized additive models: an introduction with R. CRC Press, Boca Raton, FL.

[evo13704-bib-0075] Yamaguchi, N. , A. Kitchener , C. Driscoll , and B. Nussberger . 2015 Felis silvestris. The IUCN Red List of Threatened Species 2015. Available athttp://www.iucnredlist.org.

