# Supplementary material for: Hybridization and introgression during density‐dependent range expansion: European wildcats as a case study
Source: Evolution. 2019 Mar 6;73(4):750–61. doi: 10.1111/evo.13704 (PMC6594108; doi:10.1111/evo.13704)
Supplement: Supplementary file 1 — Figure S1. Accuracy of the method to estimate the interbreeding success rate (γ). Figure S2. Effect of interbreeding success rate on the proportion of introgressed genes in a theoretical invasive and local species taken from the square world described in Fig. 2 of the main text. Figure S3. Differences in the proportion of introgressed genes of positive and negative model compare to the random model of spatial dispersal. Table S1. Parameter values for seven scenarios of an invasive species in range expansion in an area occupied by a local species with interspecific competition. Figure S4. Effect of interbreeding success rate on the proportion of introgressed genes in a theoretical invasive species taken from the square world described in Fig. 2 of the main text. Figure S5. Representation of the spatial scenario of our case study about European wildcats and domestic cats (modified from Nussberger et al. 2018). [file EVO-73-750-s001.pdf]

Evolution

Supporting information

Hybridization and introgression during density-dependent range expansion: European wildcats as a case study

Accuracy of parameter estimation

We assessed the accuracy of the estimation of the interbreeding success rate (γ) in Figure 4 of the main text, by simulating the introgression between two virtual species with a fixed values of γ. We considered 15% of admixture ($\gamma = 0.15$) as the real observed value between those species. We assumed the same parameter values used in our case study between European Wildcat and Domestic cats (Table 2).

We repeated the simulations with the software SPLATCHE2 80 times in order to estimate a 95% confidence interval of "pseudo-observed introgression" for one of the interacting species. We then performed another series of simulations but varying the value of γ between zero and 40% of admixture ($\gamma = [0, 0.4]$). We repeated the simulation for each γ 20 times.

We then performed the same GAM analysis desceribed in the methods of the main text, in order to find the most likely value of γ given this pseudo-observed confidence interval of introgression. We created a dummy variable, for which 1 and 0 represent values within or outside the pseudo-observed CI at 95%, respectively. We used this variable as response in a GAM analysis with binomial error and using the values of γ as explanatory variable. By using this GAM model, we recreated 1000 probabilities within the range 0-0.4 and caught the value that better explain the pseudo-observed data (i.e. which has the highest probability of falling within the pseudo-observed 95% CI) (e.g. Fig S1A). We repeated this analysis 100 times with different pseudo-observed data.

We finally estimated the accuracy of the estimated γ (the one with the higher probability) by comparing this value with the one fixed at the beginning of the analysis ($\gamma_{pseudo-observed} = 0.15$), as follow:

$$\left(1 - \frac{\left|\gamma_{estimated} - \gamma_{pseudo-observed}\right|}{\gamma_{pseudo-observed}}\right) 100$$

Figure S1B exposes the number of repetitions of each explored value of γ with the software SPLATCHE 2 in order to capture at least 95% of accuracy (red line). For an analysis similar to the one in the main text, a minimum of nine simulations with the same value of γ is needed to capture accurate values of γ (we used 100 repetitions for the case study exposed in the main text). This analysis shows that the estimated values of γ in our case study represent accurate estimation of the admixture between European wildcat and domestic cat.

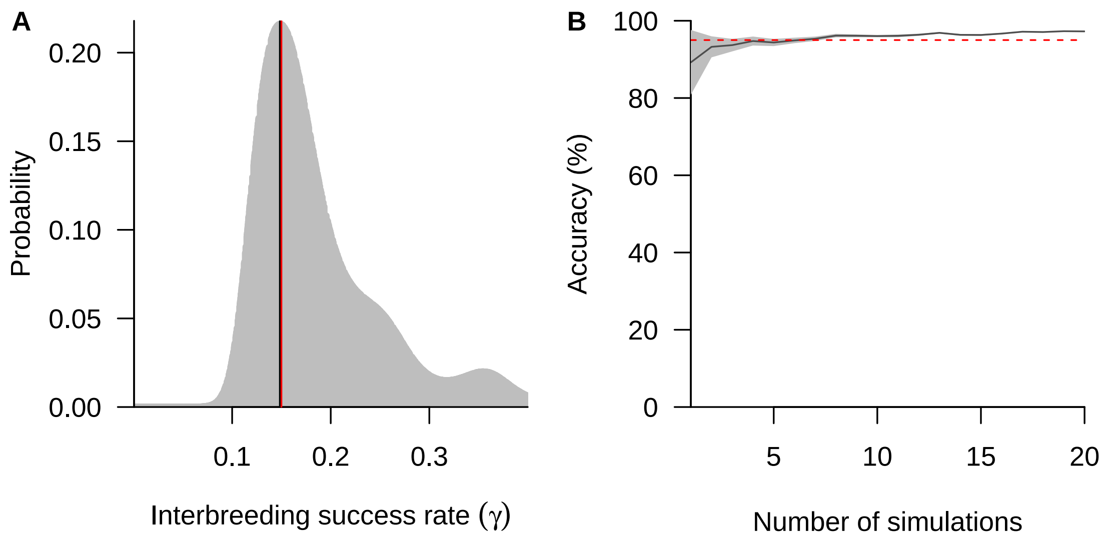

Figure S1. Accuracy of the method to estimate the interbreeding success rate (γ). We performed a series of 80 simulations with the software SPLATCHE2 in fixing the interbreeding success rate ($\gamma = 0.15$) between two virtual species under the same conditions of hybridization as our case study in the main text. It allows to generate a pseudo-observed 95% confidence interval of introgression for a known γ value. We then repeated the analysis for a range of γ values comprised between 0 and 0.4 and estimated the value with the maximum probability of explaining the pseudo-observed data (black and red line in A, respectively, see methods). The grey curve represents the density of each value of γ. We repeated this analysis for various numbers of simulations with the same γ in the software SPLATCHE2 in order to estimate the accuracy of this method (x-axis in B). The grey color in B), represent the estimation of the accuracy in a confident interval at 95%. The dotted red line delimits the 95% of accuracy for this method.

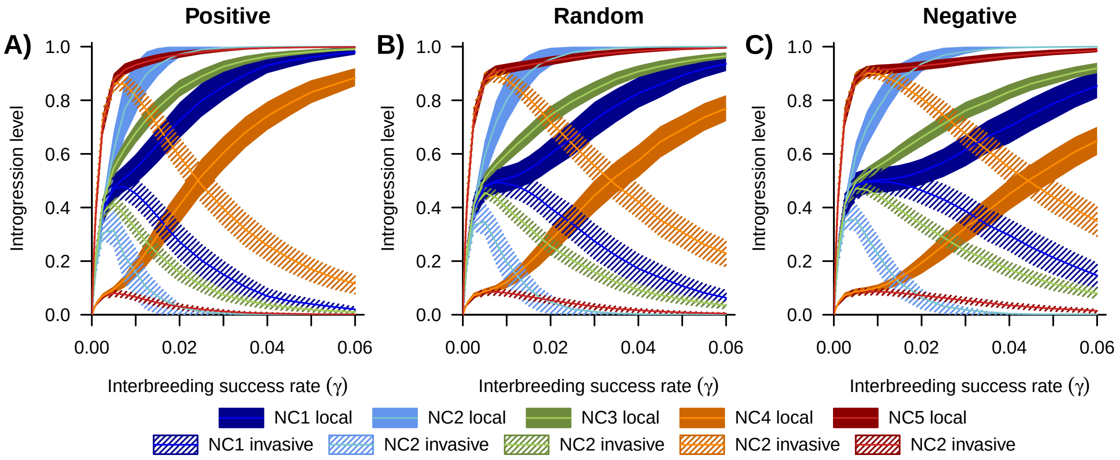

Figure S2. Effect of interbreeding success rate on the proportion of introgressed genes in a theoretical invasive and local species taken from the square world described in Fig 2 of the main text. Different models of spatial dispersal are presented: A) Positive (density-dependent), B) random (density-independent), C) Negative (density-dependent). At the onset of the invasive expansion, the local species occupied the whole area and then interact with the invasive during 1,500 years. The solid lines represent the average introgression values over 10,000 stochastic simulations. The colored areas delimit the 25%and 75% percentile of those simulated data (see Table 1 for details about different scenarios).

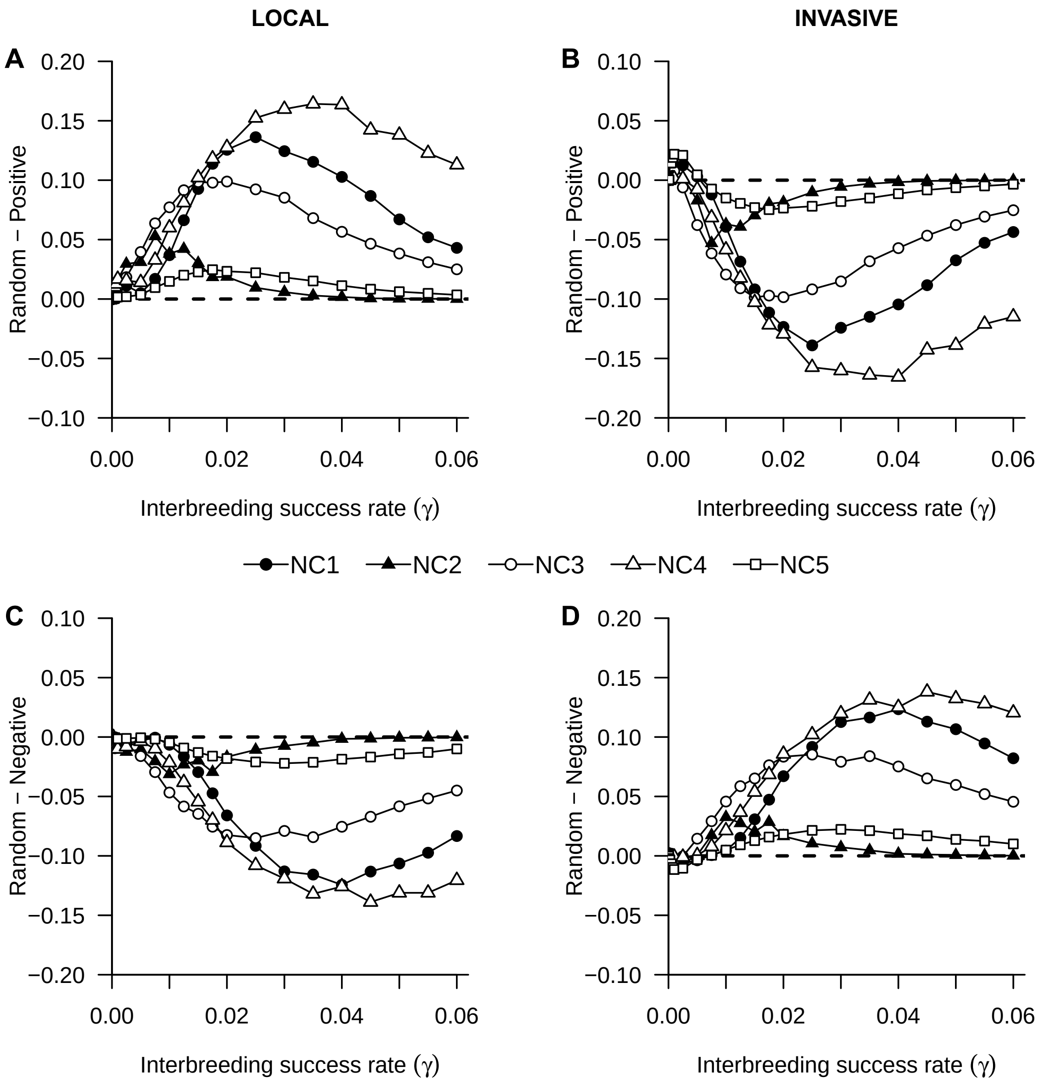

Figure S3. Differences in the proportion of introgressed genes of positive and negative model compare to the random model of spatial dispersal. At the onset of the invasive expansion, the local species occupied the whole area and then interact with the invasive during 1,500 years. A) and C) represent the differences for local organisms; B) and D) represent the differences for the invasive species. Values are average over 10,000 simulations (see Table 1 for details about different scenarios).

Table S1. Parameter values for the 7 scenarios of an invasive species in range expansion in an area occupied by a local species with interspecific competition. K is the carrying capacity and K_m the number of emigrants sent to neighbor demes when carrying capacity is reached. The intrinsic growth rate (r) is fixed to 0.5 in all scenarios.

**Scenario**	**Local species**		**Invasive species**	
	K	K_m	K	K_m
C1	50	1	500	10
C2	50	1	5000	100
C3	500	10	5000	100
C4	50	10	500	100
C5	50	10	5000	1000
C6	50	1	100	10
C7	500	10	1000	100

Range expansion of an invasive species hybridizing and competing with a local species

We simulated seven scenarios identical to those of Currat et al. (2008) of an invasive species colonizing the area of a local one, with hybridization and interspecific competition between them. The scenarios differ by carrying capacities (K) and number of migrants (K_m) sent to neighbor demes at each generation (Table S1). Interspecific competition coefficient (α_{ij}) was not fixed and assumed to be density-dependent ($\frac{N_j'}{N_i'+N_j'}$) (Currat and Excoffier, 2004). The area represents a theoretical world of 10,000 demes, in which both species are being interacting during 1,500 generations (see main text for further details about the method).

In the explored scenarios the invasive species progressively replace the local one due to competition of environmental resources and a higher carrying capacity (Fig. S4). Even if the local species is demographically extinct at the end of the simulation, their genes are still found in the invasive genome if enough interbreeding is not prevented. More than 80% of local genes are present in the invasive pool when interbreeding γ is higher than 15%. The number of local genes found in the invasive species is negatively correlated with the density of the invasive (compare scenarios C2 and C4 vs C6 and C7). In other words, the higher the invasive population density, the lower the amount of introgressed local genes in the invasive population. The level of introgression is also negatively correlated with the sent number

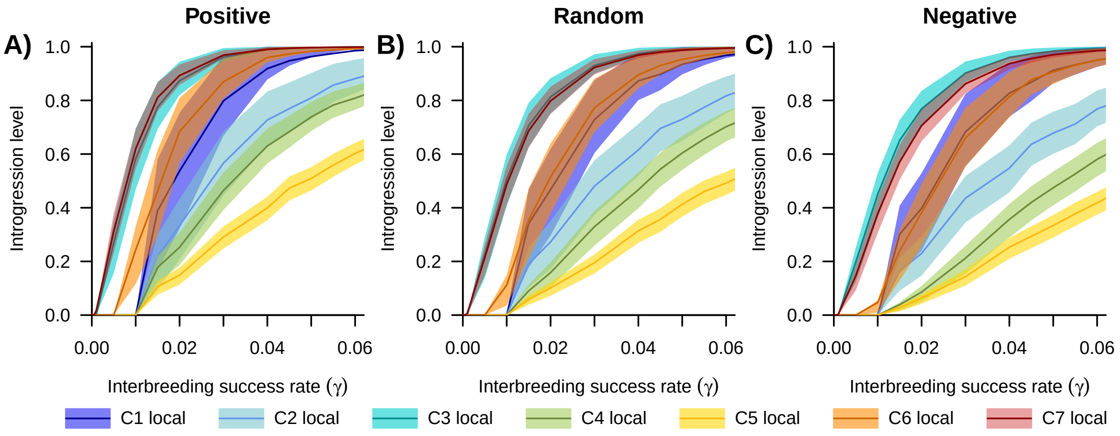

Figure S4. Effect of interbreeding success rate on the proportion of introgressed genes in a theoretical invasive species taken from the square world described in Fig 2 of the main text. Different models of spatial dispersal are presented: A) Positive (density-dependent), B) random (density-independent), C) Negative (density-dependent). At the onset of the invasive expansion, the local species occupied the whole area and then interact with the invasive during 1,500 years. Introgression values are average over 10,000 stochastic simulations. The colored areas delimit the 25% and 75% percentile of those simulated data (see Table S1 for details about different scenarios).

of migrants to neighbor demes, even if the density of the invasive is much higher (see scenario C1 and C3 vs C5) as reported by Petit and Excoffier (2009). Those observed trends are consistent in all three models of spatial dispersal but higher frequency of interbreeding (γ) is needed to find equivalent results between the positive (Fig. S4A) and the random model (Fig. S4B), while lower frequency of interbreeding γ is required for the negative model of density-dependent dispersal (Fig. S4C) to display similar introgression levels than in the random models of dispersal. The resulting differences on introgression between the density-independent (random) and the density-dependent models of spatial dispersal (positive and negative) are therefore equivalent to the ones exposed in the main text without competition (Fig. 3 and Fig. S2).

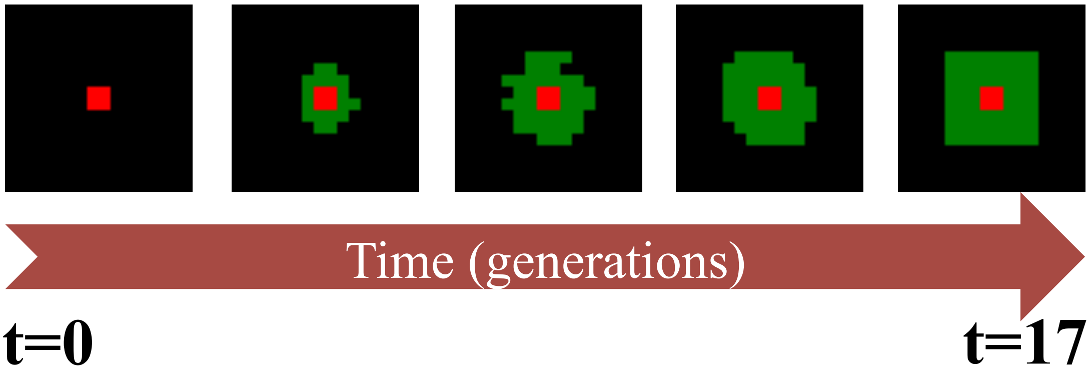

Figure S5. Representation of the spatial scenario of our case study about European wildcats and domestic cats (modified from Nussberger et al. (2018)). The red and black areas represent habitats exclusively available to European wildcats and domestic cats, respectively. The green areas are habitats used for both cats. Wildcats started a spatial range expansion in 1968. This time frame represents around 17 cat generations.

Literature Cited

Currat, M. and L. Excoffier, 2004. Modern humans did not admix with neanderthals during their range expansion into europe. Plos Biology 2:2264–2274.

Currat, M., M. Ruedi, R. J. Petit, and L. Excoffier, 2008. The hidden side of invasions: Massive introgression by local genes. Evolution 62:1908–1920.

Nussberger, B., M. Currat, C. Quilodran, N. Ponta, and L. Keller, 2018. Range expansion as an explanation for introgression in european wildcats. Biological Conservation 218:49–56.

Petit, R. J. and L. Excoffier, 2009. Gene flow and species delimitation. Trends in Ecology & Evolution 24:386–393.
